# Exosomes in inflammatory tissue injury: key pathogenic factors and promising therapeutic agents

**DOI:** 10.3389/fimmu.2026.1830034

**Published:** 2026-05-26

**Authors:** Yimeng Wang, Yihong Ye, Danni Cai, Yihan Xu, Yingping Cao

**Affiliations:** 1 Department of Clinical Laboratory, Fujian Medical University Union Hospital, Fuzhou, China; 2 Central Laboratory, Fujian Medical University Union Hospital, Fuzhou, China

**Keywords:** clinical translation, exosome, inflammation, programmed cell death, tissue injury

## Abstract

Inflammation is an essential host defense mechanism but, when excessive or chronic, can cause severe tissue damage and organ dysfunction. Exosomes, a key subtype of extracellular vesicles, have emerged as critical mediators of intercellular communication by transferring bioactive cargo and modulating inflammatory signaling. This review summarizes the roles of exosomes in inflammation-associated tissue injury, focusing on their regulation of classical inflammatory pathways and programmed cell death. We further discuss the dual functions of exosomes as pathogenic mediators and therapeutic agents, highlighting their diagnostic and therapeutic potential.

## Graphical abstract

**Figure:**
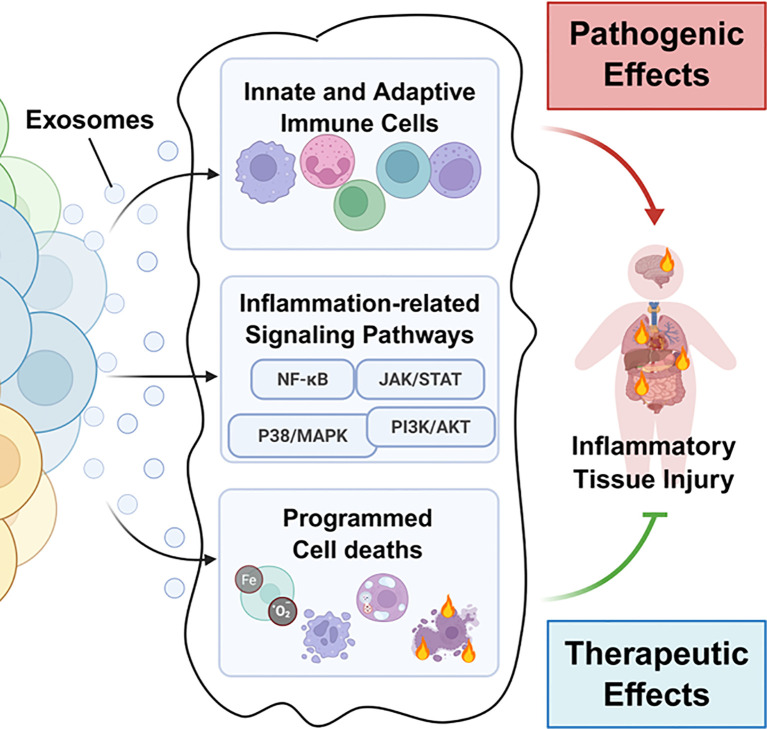

## 1 Introduction

Under normal physiological conditions, inflammation constitutes a rapid and tightly regulated host response to continuous exposure to diverse endogenous and exogenous stimuli (1). This response is initiated upon recognition of damage-associated molecular patterns (DAMPs), pathogen-associated molecular patterns (PAMPs), and oxidative stress signals. While a controlled inflammatory response is essential for pathogen clearance and tissue repair, excessive or dysregulated inflammation disrupts tissue homeostasis and contributes to the pathogenesis of a broad spectrum of human diseases (2). Notably, tissue injury itself can further amplify inflammatory responses by promoting the recruitment and activation of innate immune cells, such as neutrophils and macrophages (3). This finely orchestrated inflammation-repair cascade plays a protective role during acute tissue damage, underscoring inflammation as a complex, multiscale regulatory network (4). In contrast, severe or recurrent tissue damage often leads to sustained chronic inflammation, ultimately driving organ fibrosis, progressive functional deterioration, and, in extreme cases, organ failure or mortality (5). Consequently, maintaining organismal health critically depends on the precise regulation of inflammatory responses and the prevention of excessive or chronic inflammatory activation.

Exosomes are nano-sized extracellular vesicles that play a central role in intercellular communication by transferring bioactive molecules, including proteins, lipids, and nucleic acids, between cells. Through this cargo-mediated signaling, exosomes participate in a wide range of physiological and pathological processes. Notably, accumulating evidence indicates that exosomes exhibit dual roles: they can contribute to disease progression by facilitating pathogen dissemination, immune evasion, and inflammatory responses, while also serving as potential therapeutic tools, such as drug delivery vehicles and modulators of host immunity. This dual functionality highlights their significance as both mediators of pathogenesis and promising targets for therapeutic intervention (6, 7). Importantly, the biogenesis and molecular composition of exosomes dynamically respond to changes in the extracellular microenvironment, highlighting their potential as sensitive indicators of pathological states. In addition, advances in exosome engineering have enabled the loading of functional cargos and the modification of surface molecules to enhance targeting specificity (8). These properties support their emerging applications in disease diagnosis and targeted intervention (9, 10).

In this review, we summarize current advances in exosome-mediated intercellular communication and focus on their roles in regulating inflammatory signaling pathways and programmed cell death during inflammation-related tissue injury. We further discuss the therapeutic potential of exosome-based strategies in major inflammatory disease contexts, including ischemia–reperfusion injury, drug-induced organ damage, and sepsis-associated multi-organ failure. Throughout this review, the term “exosomes” is used to refer to small extracellular vesicles (sEVs, <200 nm) as commonly defined in the literature, unless otherwise specified.

## 2 Dysregulated inflammation and tissue injury: a vicious cycle

Tissue damage occurs when cells or tissues are harmed by internal or external harmful factors (11). The causes of damage include common factors such as mechanical trauma (12), aging (13), and bacterial or viral infections (14). Beyond the initial injury, these detrimental stimuli frequently exacerbate secondary tissue damage by disrupting immune homeostasis and compromising the structural and functional integrity of the local tissue microenvironment (15). Accumulating evidence in recent years has underscored the central role of inflammatory responses in driving this process (16). The resolution of acute inflammation is generally considered a highly coordinated and tightly regulated sequence of cellular events (17). Through a self-limiting mechanism, inflammation serves to localize and eliminate harmful stimuli, facilitate the clearance of damaged cellular components, and ultimately restore tissue homeostasis (18). The hallmark clinical manifestations of inflammation—redness, swelling, heat, pain, and impaired tissue function—primarily result from increased vascular permeability, immune cell infiltration, and the release of pro-inflammatory mediators (19).

Following tissue injury, diverse immune cell populations rapidly accumulate and orchestrate dynamic interactions that are essential for effective inflammatory resolution and tissue repair (19). However, dysregulation of immune cell functions—including excessive production of inflammatory cytokines, insufficient anti-inflammatory responses, and impaired communication between immune and stromal cells—can derail the repair process, leading to maladaptive tissue remodeling and irreversible functional impairment (20). Therefore, elucidating the molecular pathways underlying inflammation-associated tissue damage and their regulatory mechanisms is critical for advancing our understanding of disease progression and identifying novel therapeutic targets.

## 3 Exosome biogenesis and functional attributes

Exosomes are a subclass of extracellular vesicles (EVs) with a typical diameter ranging from 30 to 150 nm and are characterized by a lipid bilayer membrane (21). They are secreted by nearly all cell types, including both normal and malignant cells (22). Based on differences in size, biogenesis, and biological function, EVs are generally classified into three major categories: apoptotic bodies, microvesicles, and exosomes (23). First identified in 1983, exosomes are distinguished from other EV subtypes by their unique endosomal origin (24).

**Figure 1:**
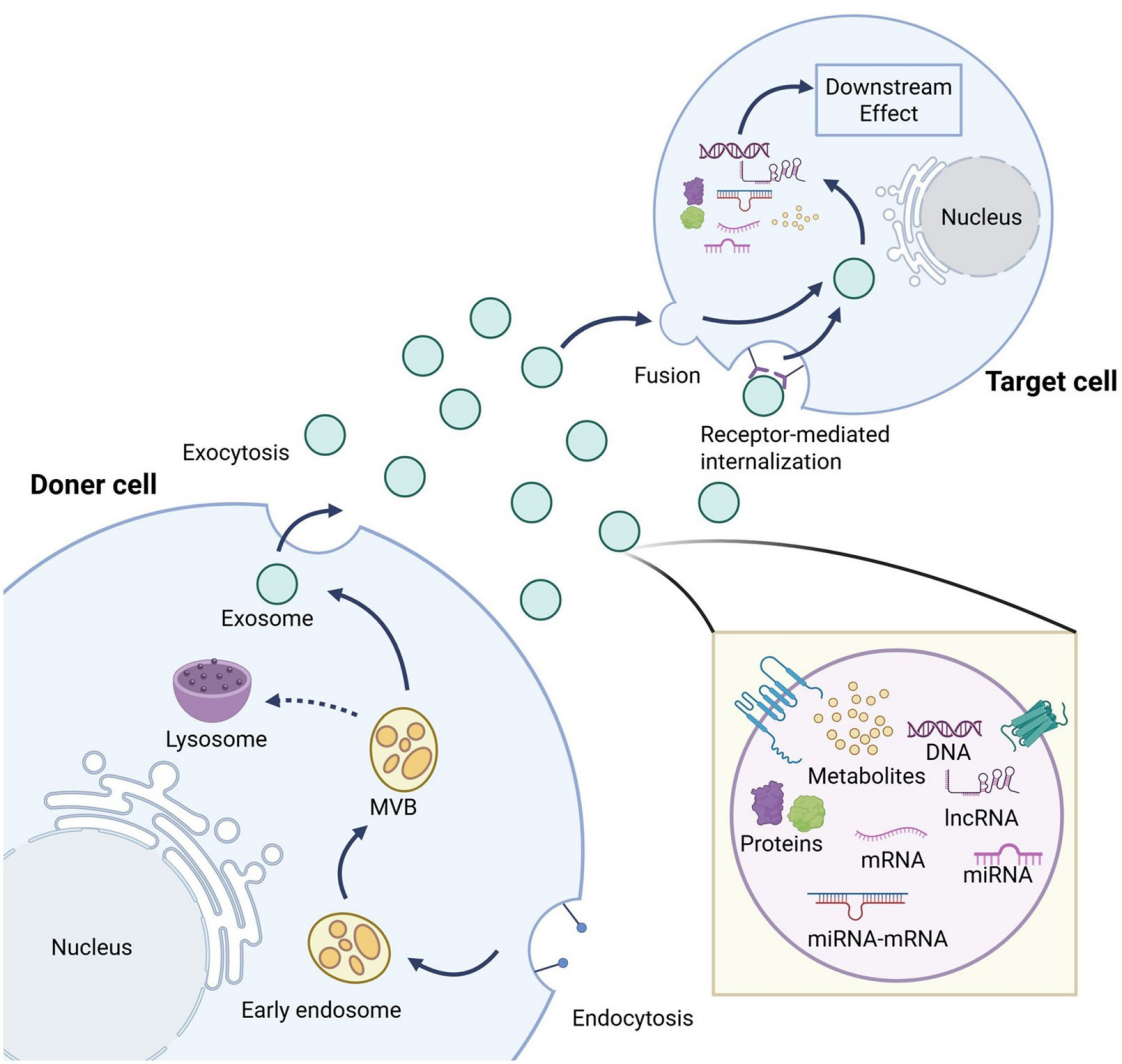
Schematic overview of exosome biogenesis, secretion, and uptake by recipient cells. Exosomes are a distinct subtype of EVs generated through the endosomal pathway. In donor cells, inward budding of the early endosomal membrane gives rise to intraluminal vesicles (ILVs), leading to the formation of multivesicular bodies (MVBs). MVBs can either fuse with lysosomes for cargo degradation or fuse with the plasma membrane to release ILVs as exosomes into the extracellular milieu. Exosomes encapsulate diverse bioactive cargos, including proteins, lipids, metabolites, and nucleic acids (e.g., microRNAs, long non-coding RNAs, and mRNAs). Following secretion, exosomes interact with recipient cells via multiple mechanisms, such as receptor-ligand interactions, direct membrane fusion, or endocytic uptake. Through the horizontal transfer of their molecular cargos, exosomes modulate gene expression, signal transduction, and cellular functions in target cells, thereby contributing to immune regulation, tissue remodeling, and the pathogenesis of various diseases, including cancer, inflammation, and neurodegenerative disorders. (Illustration created with BioRender.com).

Exosome biogenesis is initiated through endocytosis, leading to the formation of early endosomes. During endosomal maturation, inward budding of the endosomal membrane generates multiple intraluminal vesicles (ILVs), resulting in the formation of multivesicular bodies (MVBs) (25). These MVBs can subsequently fuse with the plasma membrane, releasing ILVs into the extracellular space as exosomes via exocytosis (26) (**Figure 1**). Owing to their ability to encapsulate and transport diverse bioactive cargos—including proteins, nucleic acids, and lipids (27). They are widely distributed in various biological fluids and mediate intercellular communication through both local paracrine signaling and long-range endocrine-like mechanisms, thereby modulating gene expression and cellular function in recipient cells (28). Importantly, the biological effects of exosomes are highly context-dependent, being shaped by the physiological state of donor cells as well as the local tissue microenvironment (29). Consequently, elucidating how exosomal composition and function vary across cell types and disease states is essential for understanding their roles in physiological regulation and disease pathogenesis (30). Beyond their endogenous signaling functions, exosomes have attracted increasing attention as natural delivery vehicles for therapeutic applications. Their membrane-associated and transmembrane proteins facilitate efficient cellular uptake, primarily through endocytic pathways, thereby enhancing targeting specificity and therapeutic efficacy. These properties render exosomes particularly attractive platforms for drug delivery and precision medicine strategies (31).

Multiple mechanisms have been implicated in exosome uptake by recipient cells, although many aspects of this process remain incompletely understood. While direct fusion of exosomes with the plasma membrane can occur, This mechanism is generally considered to be a minor pathway (32). Instead, endocytosis represents the predominant route of exosome internalization and encompasses several distinct processes, including phagocytosis, macropinocytosis, clathrin-mediated endocytosis, and caveolin-mediated endocytosis (33). Among these, clathrin-mediated endocytosis is one of the most extensively characterized pathways. For example, exosomes derived from PC12 tumor cells have been shown to be internalized by bone marrow mesenchymal stem cells through both clathrin-dependent and clathrin-independent mechanisms (34). Similarly, uptake of exosomes originating from gastric epithelial cells relies on integrins α6 and αX expressed on both exosomes and recipient cells and proceeds predominantly via clathrin-mediated endocytosis (35). In immune cells, particularly macrophages and other professional phagocytes, exosomes are efficiently internalized through phagocytosis, a process that is generally more effective than non-phagocytic uptake pathways (36). His mechanism plays a critical role in limiting the dissemination of pathogenic exosomes by promoting their clearance. Notably, therapeutic strategies aimed at enhancing macrophage-mediated exosome uptake have been proposed as a means to reduce tumor metastasis by depleting circulating tumor-derived exosomes and attenuating their pathological effects (37). In addition to uptake mechanisms, the endolysosomal system functions as a central intracellular trafficking network that governs the internalization, sorting, degradation, and signal transduction of exosomes (38). Importantly, this system is also intimately involved in exosome biogenesis (39). Dysregulation of endolysosomal trafficking can profoundly affect exosome production, secretion, and molecular composition (40). For instance, disruption of endolysosomal fusion through interference with the BORC–ARL8–HOPS axis has been shown to enhance exosome secretion by preventing the delivery of intraluminal vesicles to lysosomes for degradation (40).

## 4 Exosome-mediated regulation of immune cells in inflammation

**Figure 2:**
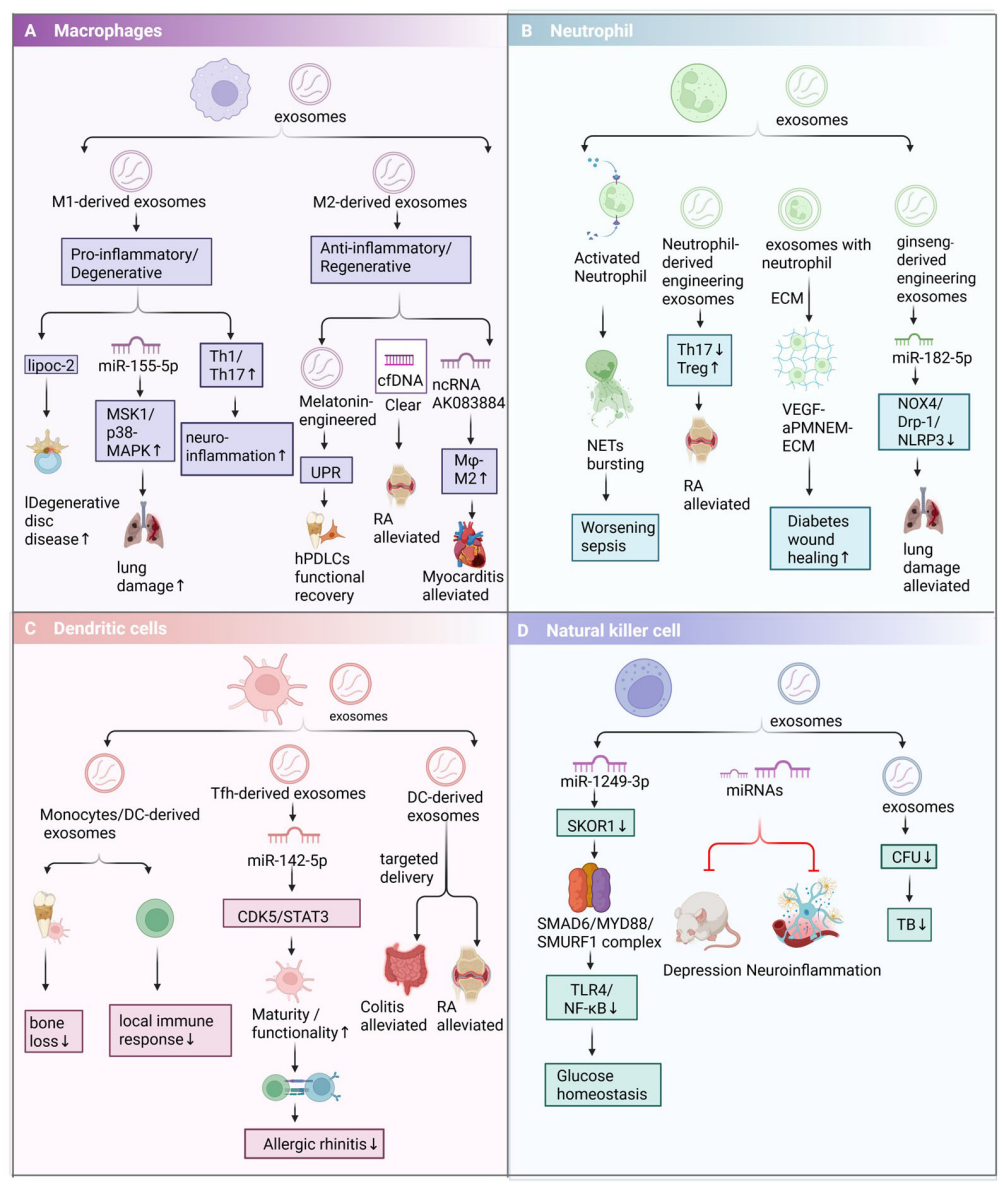
Exosomes derived from immune cells orchestrate immune responses and modulate disease progression. Exosomes released from diverse immune cell populations—including macrophages, neutrophils, DCs, and NK cells—play critical roles in maintaining immune homeostasis and shaping disease outcomes by delivering bioactive cargos such as microRNAs and proteins. **(A)** Macrophages. Exosomes derived from M1-polarized macrophages amplify pro-inflammatory signaling pathways (e.g., MSK1/p38 MAPK), thereby exacerbating tissue injury in conditions such as acute lung injury and intervertebral disc degeneration. In contrast, exosomes released from M2 macrophages or engineered macrophage-derived exosomes (e.g., loaded with melatonin or the non-coding RNA AK083884) promote anti-inflammatory responses, enhance the recovery of human periodontal ligament cells (HPDLCs), and alleviate inflammatory diseases, including rheumatoid arthritis and myocarditis. **(B)** Neutrophils. Exosomes released from activated neutrophils regulate immune balance by modulating the Th17/Treg axis and can contribute to the progression of inflammatory diseases such as rheumatoid arthritis. Conversely, engineered neutrophil-derived exosomes or neutrophil membrane-engineered ginseng-derived exosomes carrying miR-182-5p attenuate inflammation and tissue injury by targeting the NOX4/Drp1/NLRP3 signaling pathway, thereby promoting wound healing and alleviating lung injury. **(C)** Dendritic cells. Exosomes derived from monocyte-derived or mature DCs modulate local immune responses and attenuate bone loss in inflammatory conditions. In contrast, follicular helper T cell-derived exosomes enriched in miR-142-5p regulate DC maturation through the CDK5/STAT3 signaling axis, thereby mitigating allergic rhinitis. In addition, DC-derived exosomes have demonstrated potential as targeted delivery vehicles for therapeutic intervention in inflammatory diseases such as colitis and rheumatoid arthritis. **(D)** Natural killer cells. NK cell–derived exosomes deliver functional microRNAs, including miR-1249-3p, which target SKOR1 and the SMAD6/MYD88/SMURF1 complex to regulate TLR4/NF-κB signaling, glucose homeostasis, and neuroinflammatory responses. Moreover, NK cell-derived exosomes exhibit antibacterial and antidepressant effects, highlighting their multifunctional roles in immune defense and immunometabolic regulation.(Illustration created with BioRender.com.).

Inflammatory diseases are initiated and sustained by highly interconnected immunoregulatory networks. The immunomodulatory effects of exosomes are largely dictated by their cellular origin, molecular cargo, and context-dependent interactions with surrounding immune components. Consequently, exosomes derived from distinct sources may exert either pro-inflammatory or anti-inflammatory actions—even within the same pathological microenvironment (41). During inflammation, exosomes can deliver bioactive cargos to injured tissues with spatiotemporal specificity, thereby providing opportunities for targeted therapeutic intervention (42). Moreover, by mediating intercellular communication, exosomes coordinate the recruitment of immune cells and signaling mediators, reshape intracellular signaling programs, and facilitate tissue repair and regeneration (43). In this section, we summarize current evidence on how exosomes regulate the functions of key immune cell populations involved in inflammation (**Figure 2**), with an emphasis on their dual roles in amplifying inflammatory cascades and promoting the resolution phase.

### 4.1 Macrophage-derived exosomes: orchestrators of polarization and inflammation

In response to dynamic changes in the extracellular microenvironment, macrophages undergo functional polarization into distinct phenotypic subsets, classically described as classically activated (M1) and alternatively activated (M2) macrophages (44). M1 macrophages are characterized by robust pro-inflammatory activity, whereas M2 macrophages predominantly exert anti-inflammatory effects and contribute to tissue repair and remodeling (45). Under physiological conditions, moderately activated M1 macrophages play an essential protective role by exerting cytotoxic functions that facilitate pathogen clearance and the elimination of damaged or aberrant cells within the immune microenvironment (46). However, excessive or sustained M1 polarization can result in collateral damage to surrounding healthy tissues and immune cells, ultimately driving chronic inflammation and tissue injury (47). Thus, the maintenance of immune homeostasis critically depends on the precise balance between M1 and M2 macrophage activation states.

Accumulating evidence has highlighted the pathogenic significance of exosomes derived from M1-polarized macrophages (**Figure 2A**). For instance, M1 macrophage–derived exosomes have been shown to deliver lipocalin-2, thereby accelerating nucleus pulposus cell senescence and exacerbating intervertebral disc degeneration (48). In Guillain-Barré syndrome, exosomes released from M1 macrophages aggravate experimental autoimmune neuritis by enhancing Th1 and Th17 immune responses (49). Furthermore, macrophage-derived exosomal miR-155-5p has been reported to propagate M1 polarization in lung tissue through activation of the MSK1/p38 MAPK signaling axis, resulting in amplified inflammatory cascades and accelerated *Klebsiella pneumoniae*–induced acute lung injury (50).

For instance, melatonin-engineered M2 macrophage–derived exosomes have been shown to attenuate excessive endoplasmic reticulum stress and suppress activation of the unfolded protein response (UPR), thereby restoring the osteogenic and osteoinductive capacities of inflammatory human periodontal ligament cells (HPDLCs). These findings underscore the therapeutic potential of M2 macrophage–derived exosomes in inflammatory periodontal tissue regeneration (51). In rheumatoid arthritis, M2 macrophage–derived exosomes have been exploited as an immunomodulatory platform capable of scavenging circulating cell-free DNA (cfDNA), leading to effective suppression of inflammatory responses and preservation of joint integrity (52). Similarly, in viral myocarditis, exogenously administered M2 macrophage–derived exosomes deliver the long non-coding RNA AK083884 to recipient immune cells, promoting endogenous macrophage polarization toward the M2 phenotype through metabolic reprogramming. This process effectively mitigates myocardial inflammation and highlights the potential of M2 macrophage–derived exosomes as a cell-free therapeutic strategy for inflammatory cardiac diseases (53).

### 4.2 Neutrophil-derived exosomes: regulators of innate inflammatory responses

Neutrophils are among the earliest immune cells recruited to sites of tissue injury and serve as key effectors of the innate immune response (54, 55). Owing to heterogeneity in local microenvironments and activation cues, neutrophils release exosomes with diverse and context-dependent functional properties. These neutrophil-derived exosomes can transmit either pro-inflammatory or anti-inflammatory signals to neighboring cells, thereby fine-tuning the magnitude and outcome of inflammatory responses (**Figure 2B**).

In sepsis, exosomes released from activated neutrophils have been shown to induce neutrophil extracellular trap (NET) formation in resting neutrophils, triggering a feed-forward inflammatory cascade that exacerbates disease progression (56). Beyond their endogenous immunoregulatory roles, neutrophil-derived exosomes have been increasingly explored as therapeutic platforms. In chronic inflammatory rheumatoid arthritis, functionalized neutrophil-derived exosomes generated via a click-chemistry–based strategy preserve their intrinsic targeting capacity while enhancing structural stability, thereby restoring the Th17/Treg balance, suppressing inflammation, and alleviating joint damage (57). Conversely, neutrophil-derived exosomes have also been implicated in the resolution of inflammation. For example, exosomes containing leukotriene B4 (LTB4), released in coordination with nuclear DNA, promote the resolution of acute sterile skin inflammation in murine models (58). Similarly, in the context of chronic diabetic wound healing, extracellular matrix (ECM)–based hydrogels incorporating vascular endothelial growth factor (VEGF)-loaded activated neutrophil exosome mimetics (ApMNEMs) have been developed to accelerate angiogenesis and tissue repair (59). Moreover, in sepsis-induced lung injury, neutrophil membrane–engineered *Panax ginseng* root–derived exosomes loaded with miRNA-182-5p have been shown to alleviate lung injury by targeting the NOX4/Drp1/NLRP3 signaling axis (60).

### 4.3 Dendritic cell-derived exosomes: modulators of antigen presentation and T cell activation

As key sentinels bridging innate and adaptive immunity, dendritic cells (DCs) possess potent immunoregulatory functions, including antigen recognition and uptake, secretion of inflammatory cytokines, immune cell recruitment, and T cell activation and regulation within complex immune networks (61, 62). Recent studies have shown that exosomes from various sources can modulate DC differentiation, maturation, and function, offering new avenues to activate, suppress, or reprogram immune responses. (**Figure 2C**). For instance, exosomes derived from monocyte-derived DCs have been shown to influence the immune behavior of both DCs and T lymphocytes (T cells) *in vitro*, effectively modulating local immune responses and reducing bone loss in periodontitis (63). In allergic rhinitis, follicular helper T cell (TfH)-derived exosomes promote DC maturation and function via the miR-142-5p/CDK5/STAT3 pathway, leading to downstream activation of T and B lymphocytes (B cells) in lymphoid tissues (64). Furthermore, in murine models of colitis and rheumatoid arthritis, DC-derived exosomes have demonstrated effective drug-targeting capabilities and immunomodulatory effects, significantly alleviating local inflammation and tissue damage (65). These findings underscore the therapeutic potential of exosome-based strategies for immune modulation and disease intervention.

### 4.4 NK cell-derived exosomes: carriers of cytotoxic and regulatory signals

Natural killer (NK) cells are innate lymphocytes that play a central role in immune surveillance by exerting cytotoxic activity against virus-infected and malignant cells. They are predominantly distributed in peripheral blood, bone marrow, lymph nodes, and the spleen (66). Emerging evidence indicates that NK cell-derived exosomes (NKEVs), a subtype of extracellular vesicles, can transfer cytoplasmic and membrane components, including proteins, mRNA, and miRNA, from NK cells to virus-infected target cells, thereby mimicking the effector functions of NK cells (67). (**Figure 2D**).

In the context of obesity-associated inflammation and insulin resistance, NK cell-derived exosomal miR-1249-3p has been identified as a critical regulatory mediator. This microRNA directly targets SKOR1 and modulates the assembly of a SMAD6-MYD88-SMURF1 ternary complex, ultimately suppressing Toll-like receptor 4 (TLR4)/nuclear factor kappa B (NF-κB) signaling and contributing to the maintenance of glucose homeostasis (68). Beyond metabolic regulation, NK cell-derived exosomal microRNAs have also been implicated in neuroimmune modulation. In murine models of chronic mild stress, administration of NK cell-derived exosomes significantly alleviated depressive-like behaviors and reduced the production of pro-inflammatory cytokines by astrocytes, highlighting their potential neuromodulatory and anti-inflammatory effects (69).

In addition to regulatory nucleic acids, NK cell-derived exosomes are enriched in cytotoxic effector proteins, most notably perforin. Perforin functions as a pore-forming molecule that facilitates the release of granzyme B from endosomal compartments into the cytoplasm of recipient cells, thereby triggering apoptotic cell death. Notably, perforin is present in exceptionally high abundance within NK cell-derived exosomes, with levels reported to be several- to tens-fold higher than those of other cytotoxic proteins (70). Moreover, NK cell-derived exosomes have been shown to exert antimicrobial effects. In a mouse model of tuberculosis, these exosomes inhibited the survival of Mycobacterium tuberculosis by promoting apoptosis in infected cells (71).

## 5 Exosomes and inflammatory signaling pathways

**Figure 3:**
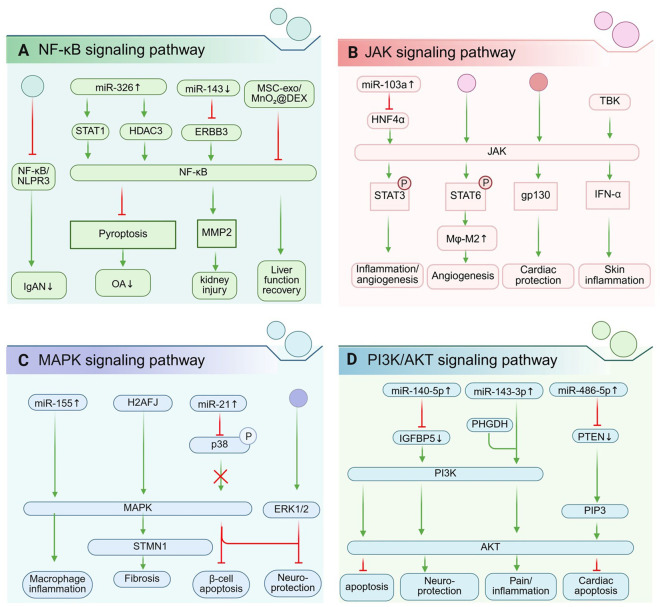
Exosome-derived microRNAs and engineered exosome systems modulate canonical inflammatory signaling pathways across disease contexts. Exosome-associated microRNAs (miRNAs), as well as engineered exosome platforms, regulate key signaling cascades implicated in inflammation, organ protection, and tissue regeneration. This schematic highlights representative pathway nodes and downstream biological outcomes modulated by exosomal cargos in diverse pathological settings **(A)** NF-κB signaling. Exosome-delivered miR-326 and miR-143 regulate inflammatory responses and tissue injury in bone/joint and renal disease contexts by targeting upstream regulators that converge on the NF-κB axis (e.g., STAT1- and ERBB3-associated signaling, respectively). In addition, engineered exosome-based interventions have been reported to mitigate inflammatory cascades and tissue damage in acute liver injury and IgA nephropathy through NF-κB–centered pathway modulation. **(B)** JAK/STAT signaling. Multiple upstream regulators and downstream effectors shape the pleiotropic functions of JAK/STAT signaling. Exosomal miR-103a suppresses HNF4α, thereby enhancing STAT3 activation to promote inflammatory responses and angiogenesis. In parallel, STAT6 activation drives macrophage polarization toward an M2 phenotype, contributing to pro-repair and angiogenic programs. Moreover, JAK1/2-dependent modulation of downstream gp130 signaling mediates cardioprotection, whereas TBK-dependent type I interferon (IFN-α) induction regulates cutaneous inflammatory responses, collectively underscoring the context-dependent roles of JAK/STAT signaling in inflammation, tissue protection, and regeneration. **(C)** MAPK signaling. Exosomal regulators (e.g., miR-155 and H2AFJ) can activate MAPK signaling to promote macrophage-driven inflammatory activation and fibrotic remodeling. Conversely, exosomal miR-21-5p suppresses p38 MAPK phosphorylation to limit β-cell apoptosis, and attenuation of p38/ERK1/2 hyperactivation has been associated with neuroprotective effects in disease models, highlighting the bidirectional and context-dependent regulation of MAPK signaling by exosomal cargos. **(D)** PI3K/AKT signaling. Exosomal miR-140-5p and miR-143-3p modulate IGFBP5- and PHGDH-associated signaling, respectively, thereby engaging PI3K/AKT cascades linked to neuroprotection and the regulation of inflammatory pain. In addition, exosomal miR-486-5p suppresses PTEN, enhancing PIP3-dependent PI3K activity and downstream AKT signaling, ultimately reducing cardiomyocyte apoptosis. (Illustration created with BioRender.com).

Within canonical immune-inflammatory signaling networks—including NF-κB, MAPK, JAK/STAT, and PI3K/AKT—exosomes have emerged as influential mediators of intercellular communication (72). By selectively packaging and transferring diverse bioactive cargos, exosomes have been reported to elicit context-dependent activation or suppression of inflammatory pathways in specific experimental settings (73)(**Figure 3**). Collectively, these properties position exosomes as versatile signaling platforms that fine-tune inflammatory responses across distinct cellular and tissue microenvironments.

### 5.1 NF-κB signaling: central regulatory axis

NF-κB is a master transcription factor ubiquitously expressed in eukaryotic cells and governs the expression of a broad array of genes involved in fundamental cellular processes, including cell survival, proliferation, and death. Most notably, NF-κB occupies a central position in the regulation of pro-inflammatory gene expression (74), thereby serving as a key molecular hub in immune and inflammatory responses. Increasing evidence suggests that the crosstalk between NF-κB signaling and exosome-mediated communication forms a complex regulatory network that integrates metabolic, oxidative, immune, and inflammatory pathways (75). (**Figure 3A**). In pathological contexts, exosomes can potentiate NF-κB–driven inflammatory signaling. For example, exosomes have been reported to accelerate the progression of diabetic nephropathy by targeting the ERBB3/NF-κB/MMP-2 axis through suppression of miR-143 (76). Similarly, in acute liver injury, a newly engineered exosome platform, MSC-EXO/MnO_2_@DEX, markedly improves liver function while concomitantly inhibiting ferroptosis, protein lactylation, and inflammatory responses, highlighting the therapeutic potential of exosome-based modulation of NF-κB activity (77). Conversely, exosomes can also exert protective effects by restraining aberrant NF-κB signaling. Bone marrow–derived mesenchymal stem cell (BMSC) exosomes deliver miR-326 to chondrocytes and cartilage tissue, where they suppress chondrocyte pyroptosis and attenuate cartilage damage by targeting HDAC3 and the STAT1/NF-κB p65 axis, ultimately alleviating osteoarthritis-associated inflammation (78). Beyond its role in transcriptional regulation, NF-κB functions as a major upstream inducer of NLRP3 inflammasome activation in immune-inflammatory diseases (79). In this regard, combined treatment with artemisinin and hydroxychloroquine in immunoglobulin A (IgA) nephropathy has been shown to enhance exosome release from human renal tubular epithelial cells (HK-2) while simultaneously suppressing NF-κB/NLRP3 signaling in human mesangial cells (HMCs) (80). This dual mechanism effectively limits inflammatory complex accumulation and mitigates renal injury.

### 5.2 JAK/STAT pathway: cytokine signal integration

JAK/STAT signaling pathway constitutes a fundamental signaling network that plays a central role in cytokine-mediated intercellular communication and immune regulation. Under acute inflammatory conditions, a broad spectrum of cytokines can rapidly activate JAK-dependent signaling cascades. Owing to the diversity of ligand–receptor interactions, distinct JAK and STAT family members can be selectively engaged, thereby conferring substantial signaling plasticity and allowing this pathway to integrate diverse external stimuli and therapeutic interventions (81). (**Figure 3B**). Accumulating evidence indicates that exosome-mediated communication critically modulates JAK/STAT signaling in a context-dependent manner. In autoimmune disease models, exosome-derived miR-103a has been shown to suppress hepatocyte nuclear factor 4α (HNF4α), leading to activation of the JAK/STAT3 axis, which in turn exacerbates inflammatory responses and promotes pathological angiogenesis in rheumatoid arthritis (82). In contrast, exosomes derived from neural stem cells exert cardioprotective effects in ischemia–reperfusion injury by modulating JAK1/2 activity and downstream gp130 signaling, thereby attenuating myocardial damage (83). Similarly, in type 2 diabetic mouse models, adipose-derived mesenchymal stem cell exosomes induce macrophage polarization toward the M2 phenotype through activation of the JAK/STAT6 pathway. This reprogramming enhances macrophage proliferation, migration, and adhesion, suppresses apoptosis, and promotes angiogenesis and revascularization in ischemic limbs (84). Conversely, exosomes derived from dermal fibroblasts of patients with systemic sclerosis have been shown to trigger type I interferon responses in keratinocytes via the TBK/JAK/STAT signaling axis, highlighting their pathogenic role in driving aberrant immune activation (85). Collectively, these findings underscore the complexity and versatility of exosome-mediated regulation of JAK/STAT signaling across diverse disease contexts. This intricacy has stimulated increasing efforts to delineate the precise molecular mechanisms underlying JAK/STAT-driven pathology and to identify actionable therapeutic targets within this signaling network.

### 5.3 MAPK pathway: stress response and tissue remodeling

Mitogen-activated protein kinases (MAPKs) constitute a highly conserved family of signaling pathways that are rapidly activated by diverse extracellular stimuli during the early phases of injury-induced inflammation. MAPK signaling orchestrates a broad spectrum of cellular responses, including proliferation, differentiation, stress adaptation, and inflammatory activation (86). Canonically, MAPKs are classified into three major subfamilies: extracellular signal-regulated kinases (ERKs), c-Jun N-terminal kinases/stress-activated protein kinases (JNK/SAPK), and p38 MAPKs (87). Dysregulation of MAPK signaling has been closely implicated in the pathogenesis of multiple inflammatory and degenerative diseases. Emerging evidence indicates that exosome-mediated intercellular communication represents a critical mechanism by which MAPK signaling is modulated in both pathological and reparative contexts. In liver fibrosis, hepatocyte-derived exosomes deliver H2AFJ to hepatic stellate cells, thereby activating the MAPK/STMN1 axis and promoting fibrogenic responses (88). Similarly, during cardiac hypertrophy, exosomes released from hypertrophic cardiomyocytes activate miR-155-dependent MAPK signaling in macrophages, leading to amplified inflammatory activation (89). Conversely, exosomes can exert protective effects by restraining aberrant MAPK activation. (**Figure 3C**). For instance, human umbilical cord blood–derived exosomes attenuate dopaminergic neuron injury in Parkinson’s disease by suppressing hyperphosphorylation of p38 MAPK and ERK1/2 through transcriptional regulation of HspB1 and Ppef2 (90). In diabetic settings, mesenchymal stem cell (MSC)–derived exosomes protect pancreatic β-cells from hypoxia-induced apoptosis by alleviating endoplasmic reticulum stress and inhibiting p38 MAPK phosphorylation via miR-21 (91). Importantly, MAPK signaling does not function in isolation. Increasing evidence highlights extensive crosstalk between MAPK and other inflammatory pathways. For example, MSC-derived exosomes have been reported to promote facial nerve regeneration by simultaneously targeting the p38 MAPK and NF-κB pathways, thereby modulating the macrophage M1/M2 polarization balance (75). These findings underscore the importance of integrated pathway regulation in achieving effective and durable therapeutic outcomes.

### 5.4 PI3K/AKT pathway: crosstalk and therapeutic implications

PI3K/Akt signaling pathway is a central regulator of diverse pathophysiological processes, including cell survival, proliferation, metabolism, and angiogenesis (92). Notably, the PTEN/PI3K/AKT/mTOR axis exhibits extensive crosstalk with multiple inflammatory signaling networks, such as MAPK, NF-κB, JNK, and WNT pathways, positioning PI3K/AKT as a key integrative node in inflammatory regulation (93). Elucidating these complex interactions is critical for the rational design of multi-target therapeutic strategies. (**Figure 3D**). In inflammatory periodontal disease, exosomes released from periodontal ligament stem cells transport miR-143-3p, which activates the PI3K/AKT/NF-κB signaling axis in M1 macrophages, thereby exacerbating inflammatory pain responses (72). In contrast, multiple studies have highlighted the therapeutic potential of exosome-mediated modulation of PI3K/AKT signaling. For example, exosomes enriched in miR-140-5p alleviate neuronal damage and improve neurological outcomes following subarachnoid hemorrhage by regulating IGFBP5-mediated PI3K/AKT signaling (94). Similarly, a recent strategy involving intralymphatic injection of KLF2-engineered exosomes demonstrated robust cardioprotective effects in murine models of myocardial ischemia–reperfusion injury (95). This approach delivers miR-486-5p to target the PTEN-PI3K/AKT pathway, thereby suppressing cardiomyocyte apoptosis. Notably, plant-derived exosomes have also emerged as promising therapeutic agents. In chronic inflammatory periodontitis, garlic-derived exosomes exert anti-inflammatory and antioxidant effects by modulating the PHGDH/PI3K/AKT pathway, ultimately reducing lipopolysaccharide-induced oxidative stress and pro-inflammatory mediator expression (96).

## 6 Exosomes and inflammatory programmed cell death

**Figure 4:**
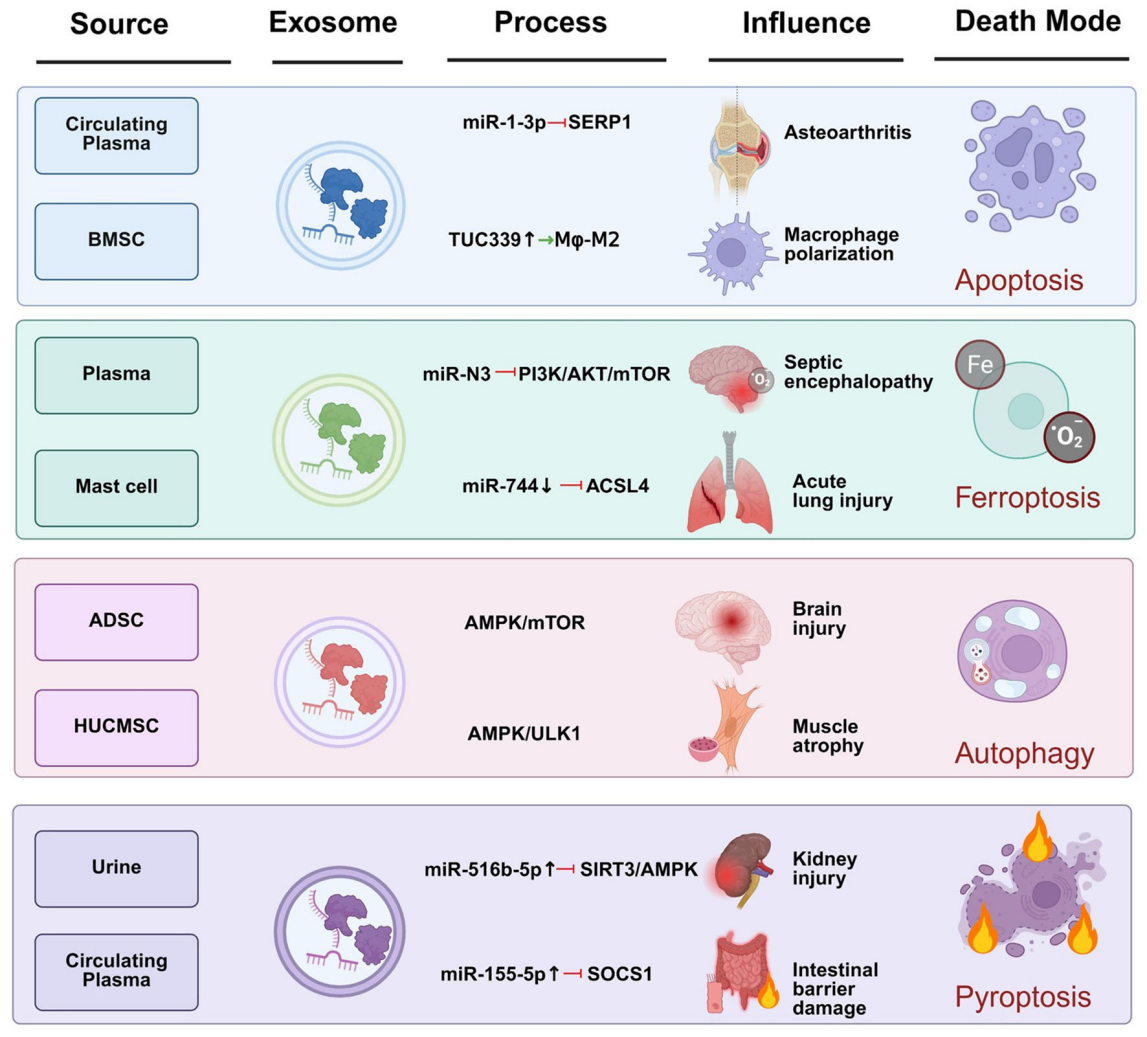
Exosome-derived regulatory cargos orchestrate multiple forms of programmed cell death across tissue-specific injury models. In apoptosis-related mechanisms, circulating plasma-derived exosomal miR-1-3p regulates SERP1 expression, thereby promoting chondrocyte apoptosis in osteoarthritis, whereas BMSC exosomes modulate TUC339 expression to influence macrophage polarization toward the anti-inflammatory M2 phenotype. Ferroptosis is primarily regulated by immune cell–derived exosomes. Mast cell–derived exosomes enriched in miR-N3 target the PI3K/AKT/mTOR pathway in sepsis-associated encephalopathy, while reduced levels of miR-744 in mast cell–derived exosomes promote lipid peroxidation and ferroptotic injury in acute lung injury through activation of ACSL4. Autophagy is modulated by exosomes derived from ADSCs and human umbilical cord–derived mesenchymal stem cells (HUC-MSCs), which activate the AMPK/mTOR and AMPK/ULK1 signaling pathways, respectively, thereby conferring neuroprotection in brain injury models and preserving muscle integrity in myopathic conditions. Pyroptosis is driven by exosome-mediated inflammasome activation. Urinary exosomal miR-516b-5p exacerbates disease progression by suppressing the SIRT3/AMPK axis, whereas plasma-derived exosomal miR-155-5p aggravates intestinal barrier dysfunction by downregulating SOCS1 and activating NLRP3 inflammasome signaling.

Historically, cell death was primarily regarded as a beneficial process that facilitated the removal of outdated, damaged, or infected cells during biological evolution, thereby maintaining internal homeostasis (97). However, similar to immune dysregulation in inflammatory diseases, imbalanced cell death signaling, either excessive or insufficient, can result in serious pathological conditions such as cancer, chronic infections, and autoimmune disorders (98). Traditionally, cell death has been categorized into two main types: accidental cell death and programmed cell death (99). This discussion focuses on programmed cell death, an active, gene-regulated process that occurs in a highly controlled and orderly manner. It encompasses several distinct pathways, including apoptosis, pyroptosis, autophagy, and ferroptosis (**Figure 4**).

### 6.1 Pyroptosis: exosome-mediated inflammatory amplification

Pyroptosis is a pro-inflammatory form of regulated cell death characterized by the formation of membrane pores, resulting in cellular swelling, membrane rupture, and the massive release of intracellular contents, including inflammatory cytokines (100). This process is primarily initiated by cysteine-dependent aspartate-specific proteases (caspases), including caspase-1, -3, -4, -5 (caspase-11 in mice), -6, -8, and -9. Upon activation, these caspases cleave members of the gasdermin (GSDM) superfamily, thereby triggering pore formation and driving cells toward pyroptotic death (101). Among the GSDM family, GSDMD-mediated pyroptosis is most closely linked to inflammatory disorders and is therefore considered to have predominant pathological relevance (102).

Emerging evidence indicates that exosomes are intricately integrated into caspase-dependent signaling networks, forming a highly coordinated regulatory axis that governs the initiation, modulation, and propagation of pyroptosis. Activation of caspase pathways not only alters the quantity of exosomes released but also profoundly reshapes their molecular cargo. For instance, during caspase-8–dependent inflammasome activation, GSDMD-facilitated by the chaperone proteins Cdc37 and Hsp90—recruits the E3 ubiquitin ligase NEDD4 to catalyze polyubiquitination of pro–interleukin-1β (pro-IL-1β). This modification promotes the sorting of IL-1β into secretory vesicles and facilitates the release of IL-1β-enriched exosomes (103). In inflammatory disease settings, exosome-mediated induction of pyroptosis in recipient cells has emerged as a particularly striking pathogenic mechanism. In murine models of acute pancreatitis, circulating plasma exosomes are highly enriched in miR-155-5p (104). Transfer of these exosomes to intestinal epithelial cells suppresses SOCS1 expression, activates the NLRP3 inflammasome, and ultimately induces pyroptosis, leading to disruption of intestinal barrier integrity (104). Similarly, urinary exosomes have been shown to accelerate the progression of diabetic kidney disease by activating NLRP3 inflammasome signaling through miR-516b-5p-mediated suppression of the SIRT3/AMPK pathway (105). Beyond nucleic acid–mediated regulation, host-derived extracellular vesicles can also capture circulating lipopolysaccharide (LPS) and deliver it directly into the cytosol of bone marrow-derived cells and endothelial cells, thereby triggering non-canonical inflammasome activation (106). Notably, recent studies further demonstrate that exosomes released from pyroptotic cells can transmit functional pore-forming GSDMD-N fragments to neighboring bystander cells, thereby propagating pyroptosis in a paracrine manner (107). Collectively, these findings highlight the diverse and multifaceted roles of exosomes as key regulators and amplifiers of pyroptotic cell death during inflammatory disease progression (**Figure 4**).

### 6.2 Ferroptosis: lipid peroxidation and iron-dependent death

Ferroptosis is a distinct form of regulated cell death characterized by iron-dependent lipid peroxidation, which ultimately leads to membrane damage and cellular demise (108). Under conditions of oxidative or metabolic stress, donor cells markedly increase exosome release, raising the possibility that exosomes may either function as protective mediators to restore redox homeostasis or act as vectors that propagate ferroptotic signals between cells (109–112). Accumulating evidence supports a context-dependent role of exosomes in ferroptosis regulation. For example, exosomal miRNA Novel-3 derived from macrophage-origin foam cells has been shown to exacerbate neuroinflammation and ferroptosis in ischemic stroke by targeting microglia and suppressing the PI3K/AKT/mTOR signaling pathway, thereby establishing a mechanistic link between atherosclerosis and stroke (109). In acute respiratory distress syndrome (ARDS)–associated lung injury, reduced levels of miR-744 in mast cell–derived exosomes enhance ferroptosis and inflammatory responses in bronchial epithelial cells by dysregulating key ferroptosis-related genes, including ACSL4, ALOX15, and GPX4 (110). Conversely, exosomes derived from MSCs exert pronounced protective effects against ferroptosis. These exosomes preserve the expression of the cystine/glutamate antiporter SLC7A11 and suppress pro-oxidant enzymes, thereby maintaining intracellular redox balance (113). Similarly, adipose-derived stem cell (ADSC) exosomes have been reported to attenuate sepsis-induced acute lung injury by delivering regulatory cargos that inhibit Keap1 and activate the Nrf2/GPX4 axis, ultimately suppressing ferroptosis in pulmonary microvascular endothelial cells (114). Collectively, these findings highlight ferroptosis as a promising therapeutic target in acute lung injury and suggest that modulating exosome-mediated cargo delivery represents a novel strategy for redox-based intervention (**Figure 4**).

### 6.3 Apoptosis: exosome regulation in non-inflammatory cell death

Apoptosis is a highly conserved, non-inflammatory form of programmed cell death that is initiated through either the intrinsic (mitochondrial) or extrinsic (death receptor-mediated) pathways (115). Similar to pyroptosis, apoptosis is caspase-dependent, with caspase-3 serving as the central executioner enzyme. Increasing evidence indicates that exosomes play important regulatory roles in apoptotic signaling by modulating both cell survival and death programs. In protective contexts, BMSC exosomes have been shown to alleviate osteoarthritis by upregulating the long non-coding RNA TUC339, thereby promoting macrophage polarization from the pro-inflammatory M1 phenotype toward the anti-inflammatory M2 phenotype. This phenotypic shift attenuates inflammatory signaling and supports chondrocyte function (116). In contrast, under pathological conditions, serum-derived exosomes from patients with sepsis have been reported to deliver miR-1-3p, which directly targets and suppresses stress-associated endoplasmic reticulum protein 1 (SERP1). This suppression inhibits endothelial cell proliferation, promotes apoptosis and cytoskeletal contraction, and increases endothelial monolayer permeability, ultimately contributing to the development of acute lung injury (ALI) (117).

Notably, strong activation of caspase-3 during apoptosis promotes the release of apoptotic exosomes (ApoEXOs), a specialized subset of extracellular vesicles that modulate the behavior of recipient cells (118). Compared with classical exosomes and microvesicles, apoptotic extracellular vesicles (ApoEVs) remain relatively undercharacterized; however, they are known to carry diverse cargos, including mitochondria, ribosomes, and nucleic acids, and participate in a wide range of physiological and pathological processes (119). Clearance of ApoEVs by macrophages or parenchymal cells occurs through caspase-3-dependent phagocytosis and represents an important mechanism for limiting inflammation (120). Paradoxically, ApoEVs released during vascular injury have also been shown to activate NF-κB signaling in endothelial cells, thereby exerting both anti-apoptotic and pro-migratory effects. This dual activity may inadvertently exacerbate endothelial dysfunction, underscoring the complex and context-dependent roles of exosome-mediated communication in apoptotic cell death and tissue remodeling (121)(**Figure 4**).

### 6.4 Autophagy: interplay between degradation and vesicle secretion

Autophagy is a highly conserved lysosome-dependent catabolic process that primarily serves a cytoprotective role by facilitating the degradation and recycling of damaged organelles, misfolded proteins, and cellular debris, thereby preserving cellular homeostasis and metabolic equilibrium (122). However, dysregulated autophagy—either excessive activation or insufficient flux—can contribute to cellular dysfunction and death. Hyperactivation of autophagy may culminate in autophagic cell death (type II programmed cell death), characterized by excessive self-digestion and loss of essential cellular components. Conversely, defective autophagy leads to the accumulation of dysfunctional mitochondria and toxic protein aggregates, exacerbating cellular stress and potentially triggering apoptosis or necrosis, depending on the magnitude and duration of injury (123).

Autophagy is tightly controlled by complex signaling networks, within which exosomes have emerged as important modulators. By delivering regulatory cargos to recipient cells, exosomes can either induce or suppress autophagic activity in a context-dependent manner (124). For example, in models of major depressive disorder, intranasal administration of adipose-derived mesenchymal stem cell exosomes alleviates depressive-like behaviors and suppresses neuroinflammation by enhancing autophagic flux through activation of the AMPK/mTOR signaling pathway (125).

Beyond its fundamental role in cellular quality control, autophagy has gained recognition as a promising therapeutic target in cancer, inflammatory, and metabolic diseases. Increasing evidence indicates that autophagy and exosome biogenesis are reciprocally regulated at multiple levels, jointly shaping both physiological adaptation and disease progression. Exosomes can promote protective autophagy to maintain tissue homeostasis, as exemplified by human umbilical cord–derived mesenchymal stem cell (HUC-MSC) exosomes, which activate the AMPK/ULK1 axis to alleviate muscle atrophy associated with diabetes and obesity (126). Conversely, autophagic activity can influence exosome production, cargo sorting, and clearance, underscoring the bidirectional nature of their interplay (127). In viral pneumonia, enhanced autophagic activity has been reported to facilitate exosome release, thereby promoting M1 macrophage polarization and recruitment and contributing to excessive cytokine production during influenza infection (128)(**Figure 4**). Collectively, these findings position autophagy-exosome interactions as a dynamic regulatory axis with context-dependent roles in tissue protection, immune activation, and disease exacerbation.

## 7 Dual roles of exosomes in acute tissue injury: from disease aggravation to regeneration

### 7.1 IR

Ischemia–reperfusion (IR) injury typically evolves through two sequential yet interrelated phases. The initial ischemic phase is characterized by abrupt interruption of blood supply, leading to oxygen deprivation, metabolic imbalance, and progressive cellular dysfunction (129). Paradoxically, restoration of blood flow during the reperfusion phase often exacerbates tissue and organ damage. During reperfusion, injured cells not only sustain intrinsic metabolic stress but also initiate robust inflammatory responses mediated by cytokine release and extensive immune cell infiltration (130). Consequently, IR injury represents a multifactorial pathological process involving oxidative stress, metabolic dysregulation, apoptosis, necrosis, and the amplification of immune–inflammatory signaling cascades (131). Clinically, IR injury underlies a broad spectrum of diseases, with acute myocardial infarction (132) and ischemic stroke (133) serving as prototypical examples. Moreover, IR injury is almost unavoidable during organ transplantation and remains a critical determinant of graft survival, postoperative recovery, and long-term clinical outcomes (134). Accumulating evidence indicates that tissue damage associated with IR injury is predominantly driven by sterile inflammation. During ischemia, cellular energy failure leads to degeneration and necrosis, whereas the reperfusion phase is frequently marked by delayed functional recovery despite re-established perfusion (135). Given the prominent contribution of inflammation to secondary tissue injury, anti-inflammatory strategies have emerged as promising therapeutic approaches, particularly in ischemic stroke (135).

Although the pathophysiological manifestations of IR injury vary among organs, exosome-based therapeutic strategies have been increasingly tailored to organ-specific injury mechanisms. In models of retinal ischemia, bone marrow-derived mesenchymal stem cell (BM-MSC) exosomes markedly improve functional recovery, suppress neuroinflammation, and attenuate retinal cell apoptosis (136). Similarly, in renal IR injury, engineered exosomes delivering an NF-κB pathway inhibitor (Exo-SRIκB) effectively reduce inflammatory mediator expression and preserve renal function (137). Neuroinflammatory disorders present additional therapeutic challenges due to the restrictive nature of the blood–brain barrier; however, exosomes have emerged as efficient carriers capable of crossing this barrier. In chronic cerebral hypoperfusion models, exosomes derived from microglia treated with the traditional Chinese medicine SINO are enriched in miR-223-3p, which suppresses NLRP3-mediated pyroptosis in neurons (138). In parallel, exosomes released from M2-polarized microglia carrying miR-124 target USP14 and significantly alleviate brain IR injury in murine models (139).

**Figure 5:**
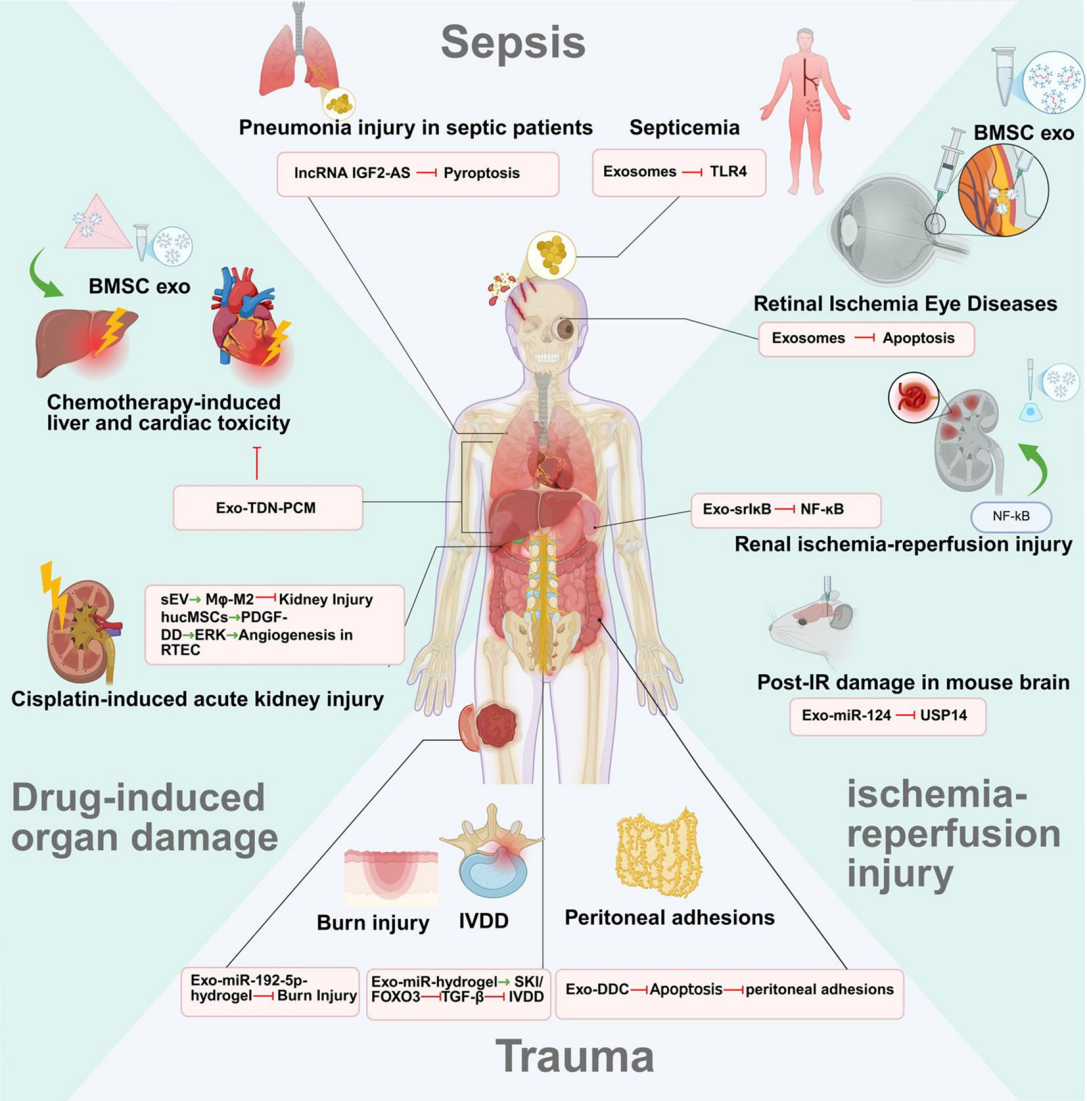
Exosome-based therapeutic strategies for injury-related diseases. This schematic summarizes the multifaceted therapeutic roles of exosome-based interventions—particularly BMSC-EXOs—in the treatment of injury-related diseases, including sepsis, drug-induced organ damage, IR injury, and trauma. In sepsis, exosomal long non-coding RNAs (e.g., IGF2-AS) modulate key inflammatory pathways by regulating pyroptosis and attenuating systemic inflammation through targeting inflammatory receptors such as TLR4. In drug-induced organ injury, exosome-mediated delivery of bioactive factors (e.g., PDGF-DD) and activation of ERK signaling, as well as engineered vesicle systems such as Exo-TDN-PCM, confer protection against nephrotoxicity and cardiotoxicity induced by chemotherapeutic agents, including cisplatin. In IR injury, exosomes serve as efficient delivery vehicles for anti-inflammatory cargos such as SRIκB and miR-124, which target NF-κB and USP14 signaling pathways to reduce neuronal and renal damage. In trauma-associated injuries—including burn wounds, intervertebral disc degeneration (IVDD), and postoperative peritoneal adhesions—exosome-loaded hydrogel systems (e.g., those delivering miR-192-5p) enhance localized exosome retention and sustained release, thereby promoting tissue regeneration, inhibiting apoptosis, and attenuating fibrotic remodeling. Collectively, this figure highlights the broad therapeutic potential of exosome-based strategies in resolving inflammation, limiting secondary tissue damage, and promoting regeneration across diverse forms of systemic injury. (Illustration created with BioRender.com).

The heart is particularly susceptible to IR injury owing to the high energetic demands of cardiomyocytes, their limited energy reserves, and minimal regenerative capacity (140). Accordingly, the therapeutic potential of mesenchymal stem cell–derived exosomes in cardiac IR injury has been extensively investigated. For example, BM-MSC–derived exosomes combined with the cardioprotective compound astragaloside promote neovascularization and suppress inflammatory responses in rat models of acute myocardial infarction (141). Moreover, miR-125a-5p–enriched MSC exosomes enhance M2 macrophage polarization, stimulate angiogenesis, and inhibit fibroblast proliferation and activation, collectively reducing cardiomyocyte apoptosis and inflammatory injury (142)(**Figure 5**).

### 7.2 Drug-induced tissue injury

In modern healthcare systems, pharmacological agents are indispensable for disease treatment and health maintenance. However, the extensive and often long-term use of medications has led to an escalating public health concern: drug-induced tissue injury. This condition refers to inflammatory and degenerative damage to cells or organs caused by drugs or their metabolites and is driven by complex and interconnected mechanisms, including oxidative stress, metabolic dysregulation, apoptosis, and immune-mediated injury (143). The liver, as the primary metabolic organ, is particularly vulnerable to drug-induced injury. It performs highly specialized biotransformation processes that convert most orally administered drugs into excretable metabolites, inevitably exposing hepatocytes to high concentrations of xenobiotics (144). Clinical evidence indicates that more than 60% of drugs requiring metabolic processing accumulate in the liver, rendering it especially susceptible to drug-induced liver injury (DILI) (145), which is now recognized as a leading cause of acute liver failure. Despite its clinical significance, DILI diagnosis currently relies largely on serum alanine aminotransferase (ALT) and aspartate aminotransferase (AST) levels, approaches that suffer from limited sensitivity, specificity, and temporal resolution (146). This conventional approach has significant limitations in terms of diagnostic accuracy and timeliness (147). Consequently, the identification of reliable and early diagnostic biomarkers has become an urgent clinical and research priority. Notably, accumulating evidence demonstrates that exosome-associated microRNAs and proteins accurately reflect disease severity and progression in liver disorders, positioning exosomes as promising non-invasive biomarkers (147). For example, circulating plasma exosomes enriched in liver-specific microRNAs, including miR-122, miR-192, and miR-155, are markedly elevated during acetaminophen-induced liver injury, highlighting their potential for early and precise diagnosis and timely clinical intervention (148).

Chemotherapy remains a cornerstone of cancer treatment; however, its lack of tumor specificity frequently results in off-target toxicity affecting multiple organs (149). This challenge has attracted increasing attention and has given rise to the emerging field of cardio-oncology (150). Doxorubicin exemplifies the “double-edged sword” nature of chemotherapeutic agents, combining potent antitumor efficacy with a high risk of cardiotoxicity that significantly compromises long-term patient outcomes (151).

In response to these limitations, engineered exosome-based strategies for tissue protection and repair have gained considerable interest. Recent studies have demonstrated that bone marrow mesenchymal stem cell–derived exosomes loaded with tetrahedral DNA nanostructures (Exo-TDN-PCM) markedly alleviate chemotherapy-induced inflammatory liver injury while simultaneously reducing cardiac toxicity, without compromising antitumor efficacy (152). These findings highlight the potential of exosome-based therapies to mitigate the adverse effects of chemotherapy while preserving its anticancer efficiency. As emerging biological drug delivery vehicles, exosomes possess inherent tissue- and organ-homing capabilities, which constitute one of their most valuable therapeutic attributes. These nanoscale vesicles enhance drug accumulation, tissue penetration, and cellular uptake at target sites (153). For instance, chitosan-modified exosomes have demonstrated preferential targeting of liver tumors while exerting pronounced cardioprotective effects, further underscoring the versatility of exosome-based therapeutic platforms (154). Beyond the liver and heart, the kidneys represent another major target of drug-induced toxicity (155). Owing to their role in blood filtration and excretion, kidneys receive approximately 20-25% of cardiac output and are exposed to high concentrations of circulating drugs and metabolites. Combined with the concentrating capacity of renal tubules, this renders the kidney the second most vulnerable organ to drug-induced injury. Cisplatin, a widely used chemotherapeutic agent known for its high efficacy, is significantly limited in clinical practice due to its severe and dose-dependent nephrotoxicity, which restricts both the dosage and duration of treatment (156). The primary mechanism of cisplatin-induced kidney injury involves the induction of renal inflammation and apoptosis in renal tubular epithelial cells (157). Recent studies highlight the therapeutic promise of exosomes in mitigating cisplatin-induced kidney injury. Small extracellular vesicles derived from human umbilical cord mesenchymal stem cells promote macrophage polarization toward the anti-inflammatory M2 phenotype, suppress pro-inflammatory cytokine production, and enhance renal repair in cisplatin-induced acute kidney injury (158). Furthermore, pretreatment of HUC-MSCs with resveratrol enhances the paracrine secretion of PDGF-DD, activating ERK signaling in renal tubular cells and promoting angiogenesis in endothelial cells (159). Collectively, these findings underscore the dual diagnostic and therapeutic potential of exosome-based strategies in drug-induced tissue injury. The remarkable efficacy, targeting capability, and translational versatility of exosome-based therapeutics herald a new era of precision medicine for the prevention and treatment of drug-induced acute organ injury.

### 7.3 Sepsis

Sepsis is a complex clinical syndrome characterized by infection-triggered immune dysregulation that culminates in inflammation-driven tissue injury and organ dysfunction (160). Its clinical course typically evolves from an early phase of excessive inflammatory activation to a subsequent stage of immune suppression, reflecting a dynamic and tightly interconnected temporal continuum (161). With growing insights into the pathophysiological roles of exosomes in sepsis, modulation of inflammatory responses remains a central therapeutic focus (162). In parallel, additional pathological processes—including coagulopathy and regulated cell death-have emerged as critical and potentially targetable contributors to disease progression (163).

During sepsis, immune dysregulation induces a hyperinflammatory state accompanied by widespread platelet activation, aggregation, and consumption (164), which profoundly disrupts hemostatic balance and can progress to disseminated intravascular coagulation (DIC) (165). Notably, the majority of circulating plasma exosomes originate from platelets, and accumulating evidence indicates that platelet-derived exosomes contribute to vascular apoptosis, myocardial dysfunction, and neutrophil extracellular trap (NET) formation during sepsis (166). These processes form a self-amplifying pathological loop that links coagulation abnormalities with inflammatory escalation, positioning platelet-derived exosomes as a critical mechanistic bridge between hemostasis and immune dysfunction.

Excessive programmed cell death further represents a major driver of sepsis-associated organ failure (167). Exosomes isolated from septic patients have been shown to induce apoptosis in endothelial and vascular smooth muscle cells, an effect attributed in part to elevated nicotinamide adenine dinucleotide phosphate (NADPH) oxidase activity within these vesicles (168). Importantly, exosomes involved in cell death regulation display marked heterogeneity in cargo composition, release dynamics, and biological function (169). For instance, mesenchymal stem cell–derived exosomes carrying the long non-coding RNA IGF2-AS attenuate pneumonia-associated sepsis by regulating endothelial pyroptosis through modulation of high-mobility group box 1 (HMGB1)–dependent nucleotide metabolism, thereby alleviating organ injury (170). These pyroptosis-derived exosomes can interact with B cells to suppress TLR4 signaling, limiting further cell death and enhancing host survival during sepsis. Collectively, these findings underscore the multifaceted and context-dependent roles of exosomes in coordinating inflammation, coagulation, and cell death during sepsis (171)(**Figure 5**).

### 7.4 Trauma

Trauma remains a leading cause of mortality among young individuals worldwide and poses a substantial burden on modern healthcare systems. Acute inflammatory injuries associated with trauma include mechanical, thermal, chemical, and post-surgical insults. When tissue damage exceeds the body’s reparative capacity, a dysregulated immune response ensues, driving sustained inflammation, tissue destruction, multiple organ dysfunction, and heightened susceptibility to secondary infections and systemic inflammatory storms (172). In this context, exosomes have emerged as promising cell-free therapeutic agents that regulate the initiation, amplification, and propagation of post-traumatic inflammation (173, 174). Despite their therapeutic promise, naturally derived exosomes face several limitations that hinder clinical translation, including limited targeting specificity, non-selective biodistribution, and rapid *in vivo* clearance (175). These challenges significantly constrain their efficacy in precision trauma management.

In recent years, the incorporation of hydrogel-based delivery systems with exosomes has emerged as an innovative strategy to overcome these obstacles, providing localized retention, sustained release, and enhanced stability of exosomes at the site of injury (176). To overcome these barriers, hydrogel-based exosome delivery systems have gained increasing attention. By encapsulating exosomes, hydrogels provide localized retention, protect vesicles from enzymatic degradation, and enable sustained, site-specific release, thereby enhancing therapeutic stability and bioavailability at injury sites (176).

Recent studies have demonstrated the translational potential of hydrogel-assisted exosome delivery across diverse trauma-related settings. Burn injuries, characterized by excessive oxidative stress, prolonged inflammation, impaired angiogenesis, and delayed re-epithelialization, represent a particularly hostile wound microenvironment (177). To address these challenges, an MXENE–GELMA hydrogel incorporating exosomes loaded with antagomiR-192-5p was developed to selectively downregulate OLFM4, a key regulator of epidermal regeneration. This targeted strategy significantly accelerated re-epithelialization and improved burn wound healing outcomes (178).

Postoperative trauma presents another major clinical challenge, with peritoneal adhesion (PA) being a frequent and debilitating complication following abdominal surgery (179). PA arises from excessive local inflammation and fibrosis, often leading to chronic pain and intestinal obstruction (180). Application of degradable exosome-loaded hydrogels at surgical sites has shown efficacy in forming localized protective barriers that modulate the wound microenvironment (181). Injectable, degradable hydrogels loaded with adipose-derived mesenchymal stem cell exosomes have been shown to form conformal protective barriers at surgical sites while enabling controlled exosome release (181). An oxidized dextran/carboxymethyl chitosan (DCC)–based exosome hydrogel system exhibited strong antioxidant capacity, modulated the inflammatory microenvironment, reduced apoptosis, promoted macrophage polarization toward the anti-inflammatory M2 phenotype, and decreased collagen deposition, thereby effectively preventing peritoneal adhesion formation and improving postoperative recovery (181).

In systemic trauma settings such as hemorrhagic shock, massive blood loss initiates complex cascades involving hemostasis, inflammation, and endocrine dysfunction, which can progress to systemic inflammatory response syndrome and multiple organ failure (182). Platelet-derived extracellular vesicles (PDEVs) have attracted attention as therapeutic agents owing to their intrinsic hemostatic, immunomodulatory, and pro-regenerative properties (183). To address rapid degradation and clearance, platelet-rich plasma–alginate dual-network hydrogels capable of sustained growth factor release (e.g., EGF and VEGF) have been developed (184). These systems significantly accelerate wound closure, enhance neovascularization, and promote cell migration, even in the presence of wound exudates, offering a robust cell-free biomaterial platform for trauma repair (184). Moreover, PDEV-loaded hydrogel dressings maintain stability and bioactivity even in the presence of wound exudates, further enhancing neovascularization and cell migration essential for tissue regeneration (185).

Beyond acute injuries, exosome–hydrogel systems have also been explored in degenerative conditions such as intervertebral disc degeneration (IVDD) (186). IVDD is characterized by progressive fibrosis of nucleus pulposus tissue, leading to chronic pain and disability (186). Mechanical stress, hypoxia, inflammation, and oxidative stress drive phenotypic transformation of nucleus pulposus cells and accelerate fibrotic remodeling (187). A targeted exosome–hydrogel platform modified with anti–fibroblast activation protein (anti-FAP) was recently developed to selectively deliver therapeutic mRNA to fibrotic NP cells (188). This approach inhibited transforming growth factor-β signaling through the SKI/FOXO3 axis, effectively attenuating fibrosis and slowing IVDD progression (188) (**Figure 5**).

### 7.5 Other tissue injury

Similar to regulated cell death, cellular senescence represents a fundamental physiological process; however, the accumulation of senescent cells profoundly compromises tissue structural integrity and functional homeostasis (189). Extensive evidence has identified multiple interconnected mechanisms linking cellular senescence to tissue damage, including diminished regenerative capacity of stem cells (190), impaired cellular differentiation (191), mitochondrial dysfunction (192), and defective autophagic flux (193). Among these processes, immune dysfunction has emerged as a central driver of aging. Persistent immune dysregulation, characterized by chronic low-grade inflammation and sustained elevation of circulating pro-inflammatory cytokines, disrupts tissue repair and compensatory mechanisms, thereby accelerating senescence-associated tissue deterioration (194, 195).

Growing evidence indicates that exosomes actively participate in the propagation of cellular senescence. In intervertebral disc degeneration, exosomes derived from senescent cartilage endplate stem cells exacerbate oxidative stress injury in nucleus pulposus cells by modulating FOXO3 signaling, thereby accelerating disc degeneration. These findings suggest that senescence-associated exosomes function not merely as biomarkers but also as active mediators of age-related tissue pathology (196).

To delay, reverse, or prevent age-associated functional decline and enable early intervention in degenerative diseases, exosomes have garnered increasing attention as cell-free regenerative therapeutics (197). Stem cell–derived exosomes, in particular, exhibit pronounced anti-aging properties across multiple tissues. For example, mesenchymal stem cell (MSC)–derived exosomes attenuate senescence of retinal pigment epithelial cells in early diabetic retinopathy by activating the PI3K/AKT–Nrf2 signaling axis (198). In parallel, a sustained-release, cell-free osteoarthritis therapy using umbilical cord MSC–derived exosomes has been developed to selectively target chondrocytes and rejuvenate aged cartilage, highlighting the translational potential of exosome-based rejuvenation strategies (199).

Beyond aging, the human body is continuously exposed to diverse microbial communities, including bacteria, viruses, fungi, and parasites, primarily through the respiratory and gastrointestinal tracts. Effective microbial clearance requires a highly coordinated innate and adaptive immune response (200). Disruption of microbial homeostasis can result in localized tissue injury or progress to systemic dysfunction and life-threatening disease states (201).

Despite the availability of numerous antibiotics and pathogen-specific therapeutics, rapid microbial evolution and the emergence of drug resistance continue to undermine treatment efficacy (202). In this context, exosomes offer unique therapeutic advantages. In addition to their modifiable surface ligands that enable precise targeting, exosomes carry endogenous antimicrobial and immunoregulatory cargos, including cytokines and regulatory RNAs. For instance, alcohol-induced maturation of dendritic cells enhances the immunomodulatory capacity of their exosomes, which can restore impaired T cell responses during hepatitis B virus infection (203). Similarly, engineered M2 macrophage–derived exosomes have been developed as antibiotic-loaded delivery vehicles that selectively home to sites of infection, demonstrating robust efficacy in the treatment of pathogen-induced pneumonia (204).

Collectively, these exosome-based bioactive delivery platforms overcome key limitations of conventional antimicrobial therapies by integrating immune modulation with targeted antimicrobial action. As such, they represent a promising and versatile strategy for combating severe infections, particularly in the era of escalating antimicrobial resistance.

## 8 Discussions

Building on the above discussion, exosome-mediated regulation of inflammatory tissue injury is best understood as an integrated and context-dependent process linking immune cell dynamics, signaling pathway activity, and programmed cell death (205). Rather than operating through isolated mechanisms, exosomal cargos—particularly microRNAs—modulate shared signaling nodes such as NF-κB, MAPK, JAK/STAT, and PI3K/AKT, thereby influencing immune cell phenotypes and downstream cell fate decisions in parallel (206).

In this framework, pro-inflammatory immune states are often associated with enhanced signaling activation, oxidative stress, and inflammasome priming, which can favor pyroptotic and ferroptotic responses (207). In contrast, anti-inflammatory or reparative contexts are generally linked to attenuated signaling activity and reduced susceptibility to stress-induced cell death. Importantly, certain forms of programmed cell death can further reshape immune and signaling responses through the release of inflammatory mediators, suggesting the presence of bidirectional feedback among these processes (208).

Taken together, current evidence indicates that exosome-mediated effects arise from interactions among immune regulation, signaling pathways, and cell death programs, with the balance among these processes likely contributing to differences in inflammatory progression and resolution across disease settings (209). However, it should be noted that most current evidence is derived from individual experimental models, and whether these mechanisms are conserved across systems remains to be fully established.

A deeper understanding of donor cell-specific exosome biogenesis, cargo selection, and functional delivery mechanisms will be essential for identifying tractable therapeutic targets and enabling rational design of exosome-based interventions. Such mechanistic insights will accelerate translation from experimental observations to clinically meaningful applications in inflammation-associated diseases. Despite rapid progress, key challenges remain, including scalable manufacturing, purification standardization, cargo consistency, potency assays, biodistribution control, and long-term safety evaluation.

Importantly, both natural and engineered exosomes are being actively explored for inflammatory tissue injury, supported by a growing number of clinical studies. Current strategies include loading exosomes with therapeutic RNAs or proteins, engineering surface molecules to enhance targeting specificity, and functionalizing vesicles with ligands to achieve cell- or tissue-selective delivery. In parallel, emerging evidence suggests that bioactive vesicles can also be obtained from alternative natural sources—such as fruits, vegetables, herbs, and milk—potentially offering accessible and scalable options to expand the exosome toolbox, although their composition, standardization, and clinical applicability require further validation.

Although this review highlights the considerable therapeutic potential of exosomes in immune-mediated inflammatory diseases, their clinical translation remains at an early and still evolving stage. From a translational perspective, several unresolved issues limit the development of exosome-based therapeutics. First, standardization remains a major bottleneck. Differences in donor cell source, culture conditions, isolation methods, purification strategies, storage, and characterization can substantially affect vesicle yield, purity, cargo composition, and biological activity (210). The MISEV2023 guidelines provide updated recommendations for extracellular vesicle production, separation, and characterization, which may help improve reproducibility across studies (211). However, these recommendations still need to be incorporated into disease-specific and clinical-grade manufacturing workflows. Second, safety evaluation requires further refinement. Although exosomes are often considered less complex than living cell therapies, their biological effects depend on heterogeneous cargos, including proteins, lipids, nucleic acids, and metabolites. These cargos may induce unintended effects, such as off-target immune modulation (212), pro-inflammatory signaling (213), coagulation-related responses (214), or unwanted tissue remodeling (215). In particular, exosomes derived from activated immune cells, tumor cells, or poorly characterized donor cells may carry pathogenic or oncogenic signals (216). Thus, long-term biodistribution, immunogenicity, toxicity, and dose-response relationships should be carefully assessed before broad clinical application. Scalable and reproducible production of exosomes remains technically challenging. Clinical-grade manufacturing requires GMP-compliant systems, including traceable cell sources, standardized culture conditions, validated purification processes, and robust quality control (217). However, current approaches are often limited by low yield, batch-to-batch variability, and the lack of mechanism-related potency assays. Future efforts should focus on bioreactor-based scale-up, scalable purification platforms, standardized release criteria, and functionally relevant potency assays, such as those assessing anti-inflammatory activity, macrophage polarization, and inflammasome inhibition (218).

Overall, exosome-based therapies represent a promising but not yet fully validated strategy for inflammatory tissue injury. Future studies should place greater emphasis on standardized manufacturing, rigorous safety assessment, clinically relevant potency assays, and well-designed randomized controlled trials. Integrating mechanistic insights with translational requirements will be essential for determining whether exosome-based interventions can be developed into safe, reproducible, and clinically meaningful therapies. It should be noted that endogenous exosome biology and engineered exosome platforms represent distinct yet closely related aspects of the field.

## 9 Conclusions

Inflammation-related diseases remain a major global health burden, driven by complex and dynamic immune dysregulation that can culminate in tissue injury and organ dysfunction. Maintaining immune homeostasis requires tightly controlled intercellular communication across immune and stromal compartments. In this context, exosomes have emerged as pivotal mediators of cell-cell signaling, coordinating physiological adaptation and pathological remodeling at cellular, tissue, and organ levels. This review highlights the bidirectional and context-dependent roles of exosomes in inflammatory tissue injury, emphasizing their capacity to both amplify inflammatory cascades and facilitate resolution and repair. Collectively, these findings position exosomes not only as informative biomarkers but also as actionable targets and therapeutic platforms for disease intervention. The major roles of exosomes in inflammatory tissue injury are summarized in **Table 1**.

**Table 1:** Integrated overview of exosome-mediated crosstalk among immune cells, signaling pathways, and programmed cell death in inflammatory tissue injury.

Disease/condition	Immune cell context	Exosome source	Key cargo	Signaling pathway	Regulatory effect	Associated cell death	Experimental model
Acute lung injury	Macrophage (M1)	M1 macrophage-derived exosomes	miR-155-5p	MSK1/p38 MAPK	Activation (pro-inflammatory)	Pyroptosis ↑	Mouse
Klebsiella pneumoniae lung injury	Macrophage	Macrophage exosomes	miR-155-5p	p38 MAPK	Activation	Inflammation ↑	Mouse
Periodontitis	Macrophage polarization (M1→M2)	Periodontal stem cell exosomes	miR-143-3p	PI3K/AKT/NF-κB	Activation	Inflammation ↑	Mouse
Diabetic ischemic limb	Macrophage (M2)	MSC-derived exosomes	–	JAK/STAT6	Activation (M2 polarization)	Apoptosis ↓	Mouse
Osteoarthritis	Macrophage (M2)	BMSC-derived exosomes	miR-326	STAT1/NF-κB	Inhibition (anti-inflammatory)	Pyroptosis ↓	Mouse
Rheumatoid arthritis	Immune cells (macrophage/DC axis)	Exosomes	miR-103a	JAK/STAT3	Activation	–	Mouse
Sepsis	Platelet–immune interaction	Platelet-derived exosomes	NADPH oxidase	ROS/NF-κB	Activation	Apoptosis ↑	Clinical + Mouse
Sepsis-associated lung injury	Macrophage/immune regulation	MSC-derived exosomes	lncRNA IGF2-AS	HMGB1-related pathways	Inhibition	Pyroptosis ↓	Mouse
Diabetic nephropathy	Renal immune microenvironment	Circulating exosomes	miR-143 ↓	ERBB3/NF-κB/MMP-2	Activation	–	Mouse
IgA nephropathy	Renal immune cells	Renal epithelial cell exosomes	–	NF-κB/NLRP3	Inhibition	Pyroptosis ↓	Cell + Mouse
Diabetic kidney disease	Renal immune cells	Urinary exosomes	miR-516b-5p	SIRT3/AMPK/NLRP3	Activation	Pyroptosis ↑	Mouse
Acute pancreatitis	Intestinal epithelial–immune axis	Plasma exosomes	miR-155-5p	SOCS1/NLRP3	Activation	Pyroptosis ↑	Mouse
Ischemia–reperfusion injury (heart)	Cardiomyocyte–immune interaction	Neural stem cell exosomes	–	JAK1/2/gp130	Inhibition	Apoptosis ↓	Mouse
Myocardial IR injury	Macrophage–cardiac interaction	Engineered exosomes	miR-486-5p	PTEN/PI3K/AKT	Activation	Apoptosis ↓	Mouse
Cardiac hypertrophy	Macrophage–cardiomyocyte crosstalk	Cardiomyocyte exosomes	miR-155	MAPK	Activation	–	Mouse
Liver fibrosis	Hepatic stellate cell interaction	Hepatocyte-derived exosomes	H2AFJ	MAPK/STMN1	Activation	–	Mouse
Parkinson’s disease	Neuroimmune interaction	Umbilical cord blood exosomes	–	p38 MAPK/ERK1/2	Inhibition	Apoptosis ↓	Mouse
Subarachnoid hemorrhage	Neuroimmune interaction	Exosomes	miR-140-5p	IGFBP5/PI3K/AKT	Activation	Apoptosis ↓	Mouse
Ischemic stroke	Microglia/foam cell interaction	Foam cell exosomes	miRNA Novel-3	PI3K/AKT/mTOR	Inhibition	Ferroptosis ↑	Mouse
ARDS	Mast cell–epithelial interaction	Mast cell-derived exosomes	miR-744 ↓	ACSL4/GPX4	Activation	Ferroptosis ↑	Mouse

## Glossary

DAMPs: damage-associated molecular patterns
PAMPs: pathogen-associated molecular patterns
EV: extracellular vesicle
ILVs: intraluminal vesicles
MVB: multivesicular body
BMSCs: bone marrow mesenchymal stem cells
MSK1: Mitogen- and Stress-Activated Protein Kinase 1
MAPK: Mitogen-Activated Protein Kinase
UPR: unfolded protein response
hPDLCs: human periodontal ligament cells
lncRNA: Long Non-Coding RNA
DCs: dendritic cells
T cells: T lymphocytes
NETs: neutrophil extracellular traps
ECM: extracellular matrix
VEGF: Vascular Endothelial Growth Factor
NLRP3: NLR Family Pyrin Domain Containing 3
NK: Natural killer
NKEVs: NK cell-derived exosomes
miRNA: microRNA
SKOR1: SKI family transcriptional corepressor 1
NF-κB: Nuclear Factor Kappa-B
JAK: Janus Kinase
STAT: Signal Transducer and Activator of Transcription
PI3K: Phosphoinositide 3-Kinase
AKT: Protein Kinase B
IgA: immunoglobulin A
HMCs: human mesangial cells
ERK: Extracellular Signal-Regulated Kinase
JNK: c-Jun N-terminal Kinase/Stress-Activated Protein Kinase
PTEN: Phosphatase and Tensin Homolog
mTOR: Mechanistic Target of Rapamycin
Caspases: Cysteine-dependent Aspartate-specific Protease
GSDM: gasdermin
LPS: Lipopolysaccharide
AMPK: AMP-activated Protein Kinase
SOCS1: Suppressor of Cytokine Signaling 1
ARDS: acute respiratory distress syndrome
ADSCs: adipose-derived stem cells
ApoExos: apoptotic exosomes
BM-MSCs: bone marrow mesenchymal stem cells
PA: peritoneal adhesion
PDEVs: Platelet-derived extracellular vesicles
HNF4α: Hepatocyte nuclear factor 4α
EGF: epidermal growth factor
VEGF: vascular endothelial growth factor
NP: nucleus pulposus
IVDD: Intervertebral Disc Degeneration
TGF-β: Transforming Growth Factor Beta
DIC: disseminated intravascular coagulation
NADPH: Nicotinamide Adenine Dinucleotide Phosphate
HMGB1: high-mobility group box 1
TLR4: Toll-like receptor 4
DILI: drug-induced liver injury
ALT: alanine aminotransferase
AST: aspartate aminotransferase
TDN: Tetrahedral DNA Nanostructures
SEVs: Small extracellular vesicles
PDGF-DD: Platelet-Derived Growth Factor-D
IR: Ischemia-reperfusion
